# Integrated solid-state nanopore platform for nanopore fabrication via dielectric breakdown, DNA-speed deceleration and noise reduction

**DOI:** 10.1038/srep31324

**Published:** 2016-08-08

**Authors:** Yusuke Goto, Itaru Yanagi, Kazuma Matsui, Takahide Yokoi, Ken-ichi Takeda

**Affiliations:** 1 Center for Technology Innovation - Healthcare, Research & Development Group, Hitachi Ltd.,1-280 Higashi-Koigakubo, Kokubunji, Tokyo 185-8601, Japan

## Abstract

The practical use of solid-state nanopores for DNA sequencing requires easy fabrication of the nanopores, reduction of the DNA movement speed and reduction of the ionic current noise. Here, we report an integrated nanopore platform with a nanobead structure that decelerates DNA movement and an insulating polyimide layer that reduces noise. To enable rapid nanopore fabrication, we introduced a controlled dielectric breakdown (CDB) process into our system. DNA translocation experiments revealed that single nanopores were created by the CDB process without sacrificing performance in reducing DNA movement speed by up to 10 μs/base or reducing noise up to 600 pA_rms_ at 1 MHz. Our platform provides the essential components for proceeding to the next step in the process of DNA sequencing.

Single-molecule detection is vital for basic research and practical applications in nanobiotechnology and nanomedicine^1^. Nanopore sensors are a promising technology for label-free single-molecule detection^2^. Nanopore technology is particularly attractive for DNA sequencing because of its potential advantages, which include long reads, low cost and high speed^3^,^4^,^5^. These promising advantages have driven the dramatic developments in practical nanopore DNA sequencing^6^,^7^.

Depending on their composition, nanopores are categorized as biological^8^,^9^,^10^,^11^,^12^ or solid-state^2^,^13^,^14^. Although significant advances towards practical sequencing have been accomplished by utilizing biological nanopores, solid-state nanopores are more promising because of their robustness and the stability of their materials. For example, biological nanopores offer low noise performance, but the fragility of the lipid bilayer membrane limits the device lifetime and the applied voltage, which contributes to the signal-to-noise ratio. By contrast, solid-state nanopores are robust under various experimental conditions, such as high applied voltage, pH and temperature^15^,^16^.

A bottleneck in achieving solid-state nanopore sequencing is the ultrafast translocation of single-stranded DNA (ssDNA) through the nanopore^17^ even when utilizing a newly developed rapid amplifier (1 MHz sampling rate)^18^. This rapid translocation prevents the collection of sufficient current data per base. To obtain more than 10 data points per base, the speed should typically be slower than 10 μs/base. Various methods have been explored in pursuit of decelerating the passage of DNA: (i) narrowing the nanopore diameter^19^,^20^, (ii) increasing the solution viscosity^21^,^22^, (iii) decreasing the temperature^15^,^23^, (iv) neutralizing the negative charge of the DNA using alternate salts (LiCl solution)^24^. Although several groups succeeded in deceleration of DNA translocation utilizing graphene^25^,^26^,^27^,^28^ or HfO_2_^29^ nanopore, from a practical point of view, silicon nitride (SiN) nanopore are more suitable for mass-production. Despite these tremendous efforts, the deceleration of the translocation speed remains insufficient for sequencing DNA based on a solid-state nanopore (especially SiN nanopores). Moreover, these methods typically alter the ionic environment around the nanopore, which often leads to smaller ionic currents and decreases the signal-to-noise ratio.

In the past three years, several research groups have successfully developed a new method involving the use of nanometre-sized 3D structures, including nanofibre meshes^30^, gel meshes^31^,^32^, and bead structures^33^, to decelerate DNA translocation. These methods are simple yet effective in slowing the passage of DNA without sacrificing the signal-to-noise ratio. We recently demonstrated that a nanometre-sized bead structure around a nanopore can decelerate DNA translocation by up to 10 μs/base^33^ even for ssDNA, the translocation speed of which is 100-fold faster than that of double-stranded DNA^20^. Although our approach has potential for realizing DNA sequencing with a solid-state nanopore, some technical challenges remain. For example, in a proof-of-concept experiment, a nanopore has been fabricated using a transmission electron microscope (TEM) beam before providing the 3D structure on the membrane. However, from a practical perspective, TEM-based nanopore fabrication faces the following issues: (i) TEM-based fabrication is not suitable for mass-production because it is time-consuming and laborious; and (ii) although the nanopore should be fabricated after providing the 3D structure for long-term storage of a device, a nanopore cannot be intrinsically fabricated by a TEM beam because the beam cannot reach the membrane due to electron-beam scattering of the structure. Therefore, an alternative nanopore fabrication method is required that is compatible with the 3D structure needed to decelerate DNA translocation.

Alternative fabrication methods utilizing controlled dielectric breakdown (CDB) have recently been proposed and demonstrated^16^,^34^,^35^,^36^. Voltage-induced dielectric breakdown events in ultrathin membranes result in the formation of single nanopores with diameters as small as 1 nm. Additionally, CDB can precisely fabricate nanopores with sub-nanometre accuracy under the appropriate conditions^37^. Because CDB is typically based on the voltage injection process, unless the ionic behaviour is inhibited, fabrication processes based on CDB are expected to be compatible with DNA-speed-decelerating structures such as beads. Waugh *et al.* reported that nanopores can be fabricated via dielectric breakdown even after coating a gel mesh on the membrane^31^. Similarly, we expected that single nanopores could be fabricated via dielectric breakdown even after coating of a bead structure on the membrane. We recently reported a new CDB process termed “multilevel pulse-voltage injection (MPVI)”^35^. Compared with the conventional CDB process, MPVI is less damaging because it utilizes pulse-voltages to create and widen the nanopore, and nanopore generation is verified at every pulse interval by measuring the current at low voltage. In the present report, we introduce a fabrication process based on MPVI to our bead-coating device to address both nanopore fabrication and deceleration of the DNA speed.

In addition to the above challenges, a nanopore system requires the reduction of the ionic current noise to improve the signal-to-noise ratio, particularly in the high-frequency range (e.g., 1 MHz). The device capacitance arising from the surface area exposed to the solution dominates the high-frequency noise^38^. To decrease the surface area of the device, an insulating layer (e.g., poly(dimethylsiloxane) or polyimide) was also integrated into our device. In the present work, we demonstrate that dielectric breakdown induced by an applied pulse-voltage can produce a single nanopore on a membrane after coating with 50-nm nanobeads while maintaining structure. We also confirm that the deceleration of ssDNA translocation is retained following MPVI-based nanopore fabrication. Furthermore, we investigated whether an insulating layer, i.e., polyimide, coated on the device simultaneously with the nanobead structure can reduce the ionic current noise.

## Results and Discussion

### Single nanopore fabrication via MPVI

**Figure 1:**
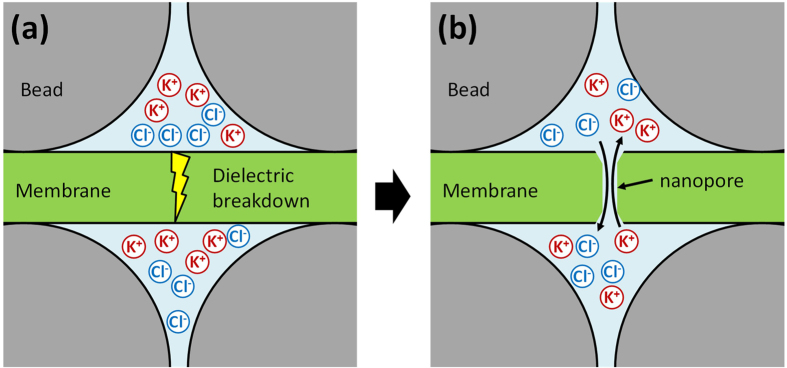
Schematic of nanopore fabrication via dielectric breakdown of an ultrathin SiN membrane with a nanometre-sized bead structure. (**a**) The electrolytes can move freely through the spaces between the nanobeads, providing ion access to the membrane. (**b**) Upon application of a trans-membrane voltage over the breakdown value to the membrane, dielectric breakdown occurred in the membrane. Because the breakdown event occurred only in the membrane, a nanopore was generated without deforming the nanobead structure.

First, we investigated the effect of the nanobead structure on the MPVI nanopore fabrication process. Waugh *et al.* reported that the gel media interface with the membrane does not prevent the dielectric breakdown process owing to the availability of ions for charge injection^31^. Similarly, we expected that the nanobead structure would have no effect on the dielectric breakdown nanopore fabrication process because the resistance of the structure can be ignored relative to that of the nanopore^33^ (Fig. 1). The behaviour of the device in DNA-decelerating media during fabrication has yet to be reported in detail, particularly in terms of whether the fabricated nanopore is truly single. Actually, when the breakdown condition is not optimized, MPVI process fails to fabricate a single nanopore (see Supplementary Figures S1 and S2). In general, single nanopore fabrication is verified by direct TEM observation. However, the DNA-decelerating structure hinders the direct observation of the nanopore on the membrane (see Supplementary Figure S3). Accordingly, prior to assessing the performance, we focused on the verification of the creation of a single nanopore by MPVI.

**Figure 2:**
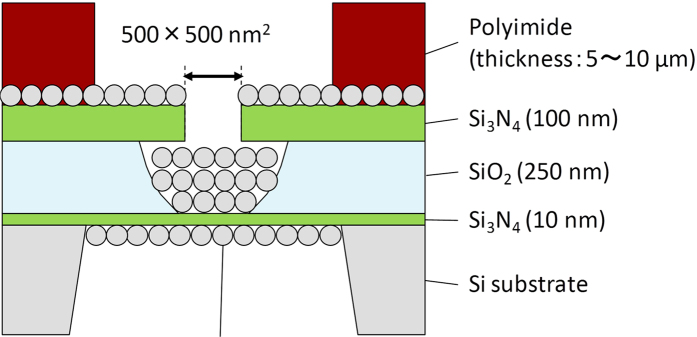
Schematic diagram of a substrate with nanobead structure and insulating layer used for nanopore fabrication via controlled dielectric breakdown. A 100-nm-thick Si_3_N_4_ layer, 250-nm-thick SiO_2_ layer, and 10-nm Si_3_N_4_ layer were deposited on the Si substrate. A square hole (500 × 500 nm^2^) was fabricated by dry etching, and the SiO_2_ layer was partially eliminated by HF etching. The bead layer was first coated over the entire surface at both sides of the substrate via the dip-coating method, and polyimide was subsequently coated on the substrate as an insulating layer.

**Figure 3:**
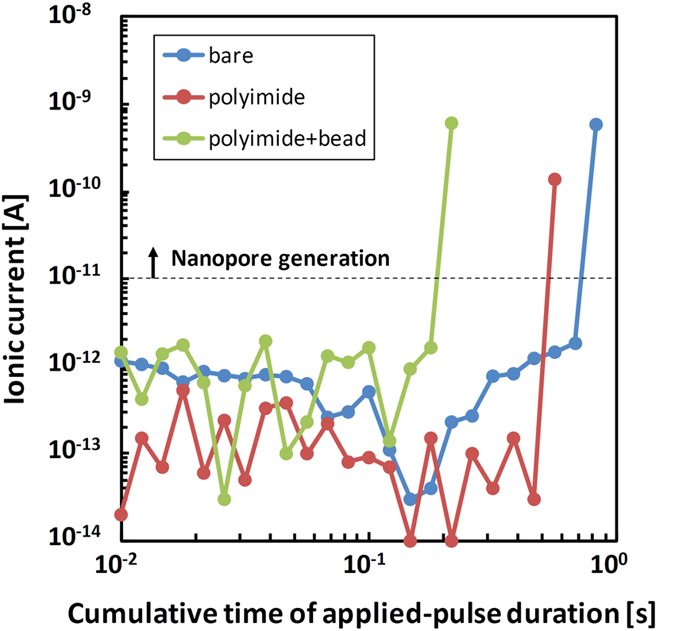
Time-dependent characteristics of the ionic current during the MPVI procedure. The dependences of the ionic currents at 0.1 V on the cumulative applied pulse duration for a bare device (blue), a polyimide-coated device (red) and a bead-polyimide-coated device (green).

Figure 2 provides a schematic diagram of the solid-state device with a layer of nanometre-sized beads on the SiN membrane from the *cis* side. The solid-state substrate for nanopore fabrication (see Supplementary Figure S4), the bead layer and the polyimide layer were prepared as previously reported^33^,^35^,^38^. Nanopore fabrication via the MPVI procedure was performed *in situ* under typical conditions (1 M KCl aqueous solution with Tris-EDTA buffer, 10 mM Tris, 1 mM EDTA, pH 7.5). A pulse-voltage from 9 V to 10 V was applied to the membrane for a maximum cumulative time of 1 s. The leakage current across the membrane at 0.1 V was monitored at every interval of the pulse. Figure 3 illustrates the leakage currents of a non-coated device (bare, blue), a polyimide-coated device (red) and a bead-polyimide-coated device (green) after the induction of a pulse-voltage. In all cases, similar dependences of the currents at a voltage of 0.1 V on the cumulative applied pulse durations were observed. After continuing feeble leakage currents through the membrane, sudden increases in the leakage currents occurred with all devices and exceeded a pre-defined threshold (10 pA, i.e., the same as the previous report^35^). This abrupt increase in the current is evidence of a typical dielectric breakdown and implies the generation of a nanopore^35^. Importantly, the breakdown voltages of the polyimide-coated devices and the bead-polyimide-coated devices (9.4 ± 0.4 V, N = 9) were nearly identical to those of bare devices (9.6 ± 0.4 V, N = 9), thus indicating that the polyimide and bead layers had no effect on the dielectric breakdown process. This behaviour is consistent with the negligible ionic resistance of the bead coating layer relative to that of the nanopore^33^ and indicates that the bead structure does not prevent the ionic path through the membrane.

**Figure 4:**
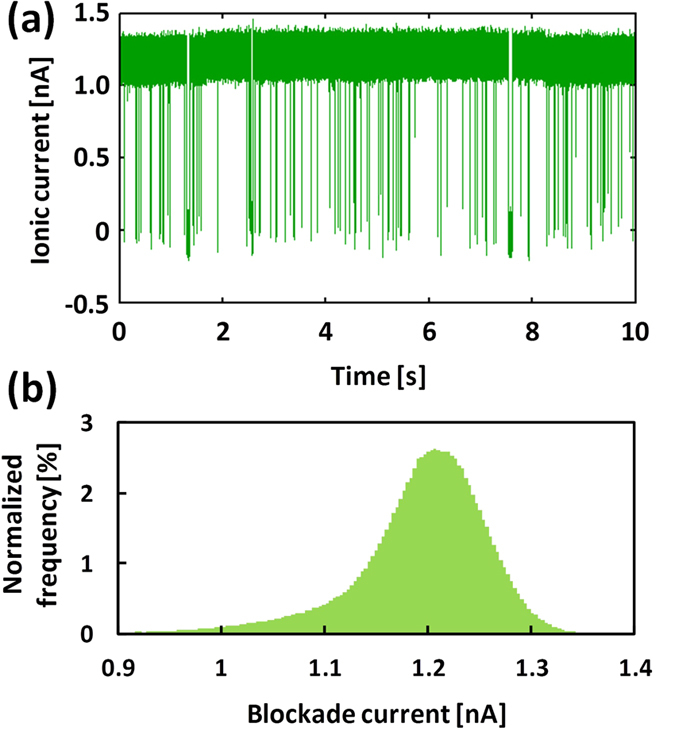
ssDNA blocking events through a nanopore fabricated via the MPVI process. (**a**) A typical time trace of the ionic current for a nanopore created via MPVI on a bead-polyimide-coated device. Blockade events for the 60-mer single-stranded poly(dA) were detected in 1 M KCl aqueous solution at 0.3 V (low-pass filtered at 100 kHz). (**b**) Normalized histogram of the blockade currents for a 60-mer single-stranded poly(dA) using the same data depicted in (**a**).

Further investigation is required to confirm the generation of a single nanopore by MPVI. The diameter of a single nanopore can be estimated from its current at a predefined applied voltage. After optimization of parameters, we confirmed that nanopore variation was quite small and MPVI process can fabricate precisely and reproducibly nanopores with sub-nanometre accuracy (see Supplementary Table S1). We examined whether the generated nanopore was indeed single by conducting ssDNA translocation experiments. Figure 4a presents typical ionic current traces for the open-state and state blocked by DNA (blocked-state) without clogging the nanopore. For a baseline ionic current of 1.2 nA at 0.3 V, the predicted diameter of a single nanopore is 1.4 nm. The full blockade phenomenon occurs when ssDNA translocates across a nanopore with a diameter of less than 1.4 nm^19^. Therefore, the mean blockade current of the ssDNA was expected to be 1.2 nA. As illustrated in Fig. 4b (blockade current histogram), the drop-down magnitude was 1.2 ± 0.05 nA and exactly equal to the open-state current level, strongly indicating that MPVI generated only a single nanopore even after polyimide and bead coating. The results also indicated that the blockade current level was not affected by the bead layer or the MPVI process.

**Figure 5:**
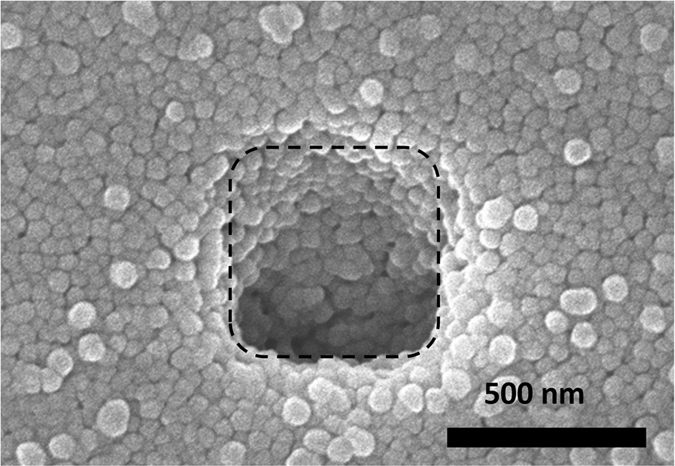
SEM observation of a bead-polyimide-coated device after the MPVI procedure. Top-view SEM image of a bead-polyimide-coated device after nanopore creation via the MPVI process. The accelerating voltage was 5.0 kV. The dashed-line indicates the square area in the SiN membrane.

After the nanopore generation and ssDNA translocation experiments, the surface condition of the device was confirmed by scanning electron microscopy (SEM) as depicted in Fig. 5. The membrane area is indicated by the dashed line. The SEM observations revealed that the close-packed bead structure was maintained without cracks even after pulse-voltage injection and DNA translocation. This closely packed state contributes to a stable nanostructure that prevents the beads from re-dispersing into the solution. Based on this observation, the maintained bead structure was expected to decrease the DNA movement speed. Overall, these results indicate that the MPVI process is suitable for nanopore fabrication on devices with nanobead structures.

### Performance in decelerating DNA movement and reducing noise

**Figure 6:**
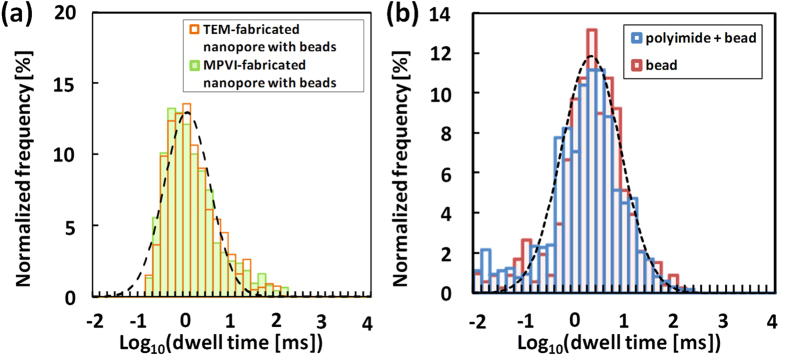
Histograms of the dwell times for ssDNA translocation. (**a**) Log-scaled histograms of the dwell times for a TEM-fabricated nanopore with beads (orange, N = 1319) and a MPVI-fabricated nanopore with beads (green, N = 1323). ssDNA (60-mer single-stranded poly(dA)) translocation was measured in 1 M KCl solution. The applied voltage was 0.3 V (low-pass filtered at 5 kHz). The nanopore diameters were 3.0 nm (orange) and 3.7 nm (green). (**b**) Log-scaled histogram of the dwell times of a bead-coated substrate (red, N = 1246) and a bead-polyimide-coated substrate (blue, N = 1712). Single nanopores in each device were fabricated via the MPVI process. Poly(dA_33_dC_33_dA_33_) was used as a ssDNA sample. The applied voltage was 0.3 V (filtered at 100 kHz). The dashed lines are curves that were fit to the log-normal distributions. The nanopore diameters were 3.4 nm (red) and 2.2 nm (blue).

The ability to control ssDNA movement speed through an MPVI-fabricated nanopore with beads was assessed with DNA translocation experiments using 60-mer single-stranded poly(dA). We previously reported that the nanosized bead structure decelerates DNA translocation by up to 10 μs/base even when using short 60-mer ssDNA^33^. Here, we investigated whether the duration of residence of ssDNA in the MPVI-fabricated nanopore with beads was identical to that in a TEM-fabricated nanopore with beads. A comparison of the durations of the two series is displayed in log-scaled histograms in Fig. 6a. These histograms are clearly similar and well fit by an identical log-normal distribution. Quantitatively, the peak dwell times of the DNA translocation through the MPVI-fabricated nanopore and the TEM-fabricated nanopore were both 16 μs/base, consistent with previously reported results^33^.

Although we used nanopores with different diameters in the translocation experiments, we consider that the effect of the nanopore size on translocation speed is negligible in the system using beads. Several researches has already reported that DNA translocation speed without any slowing-down media, such as gel or beads, depends on the diameters of SiN nanopores^13^,^20^,^39^. Especially, translocation speed of ssDNA ranged from <0.01 μs/base (3.0 − 4.0 nm) to 0.3 μs/base (1.4 − 2.0 nm)^13^,^20^,^33^. To investigate data reliability, we conducted the translocation experiment of the same 60-mer ssDNA through a nanopore without beads (see Supplementary Figure S5). Expectedly, we confirmed that translocation speed of 60-mer ssDNA was 0.3 μs/base (2.2 nm), which is consistent with the previous reported values^13^,^20^,^33^. Therefore, if the bead layer has no effect on slowing down of DNA translocation speed, the calculated dwell times of 60-mer ssDNA would be shorter than 18 μs in the experiments using bead-coated SiN nanopores with diameters from 2.3 nm to 3.7 nm. However, as shown in Fig. 6, the characteristic dwell times of SiN nanopores with beads were at least approximately 1000 μs, which are much longer than the calculated values. These results indicate that the DNA translocation process is not dominated by the interaction at the nanopore sidewall^20^,^39^ but governed by the interaction at the nanobead layer. This comparative study strongly supports that the nanobead layer can decelerate the DNA translocation speed even when integrating MPVI process.

Given that the interaction at the nanobead layer governs the translocation process, it is expected that the translocation speed of ssDNA with beads is independent on the diameters of nanopores. To verify this tendency, we conducted the translocation experiments using the several diameters of SiN nanopores with beads (see Supplementary Figures S6 and S7). Figure S7 clearly shows that translocation speed when using the substrate with beads was almost independent on the diameters of SiN nanopores (from 1.6 nm to 3.7 nm) and the average speed was 16 μs/base, while the speed using the uncoated substrate strongly depends on^20^. The results also support that ssDNA translocation is decelerated by the bead layer.

Accordingly, these results indicate that the deceleration of DNA translocation by the nanobead structure was maintained following the MPVI process and strongly suggests that nanopore fabrication via the MPVI process did not degrade the nanobead structure, in agreement with the SEM observations presented in Fig. 5. Figure 6b presents duration histograms for a bead-coated device (no polyimide) and a bead-polyimide-coated device based on DNA translocation experiments conducted with single-stranded poly(dA_33_dC_33_dA_33_). The polyimide layer would not be expected to affect the DNA translocation speed because it is located far from the nanopore membrane. As expected, the two histograms were nearly identical and were similarly well fitted by identical log-normal distributions, with a peak dwell time of 22 μs/base. Notably, the observed DNA speed is appropriate for collecting more than 10 data points per base.

**Figure 7:**
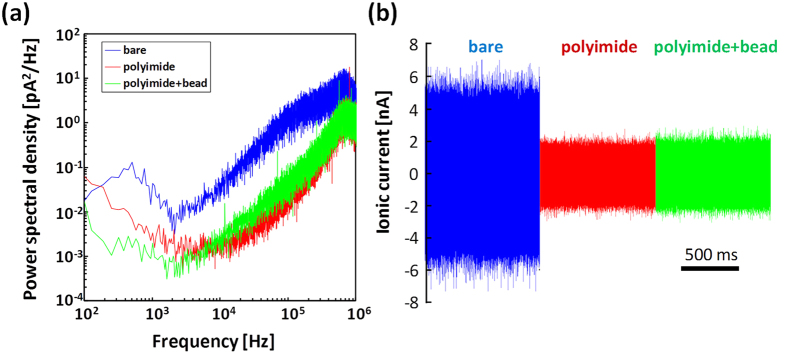
Noise characteristics of the various devices. (**a**) Current power spectral densities (PSD) of the nanopores on a bare substrate (blue), a polyimide-coated device (red) and a bead-polyimide-coated device (green). The diameters of the nanopores on each device ranged from 1 nm to 3 nm. (**b**) Typical baseline ionic current noise traces corresponding to the PSD curves described in (**a**). The ionic current noise was measured at 0 V in 1 M KCl aqueous solution using a 1-MHz high-speed amplifier VC100. The detected currents were low-pass filtered with a cut-off frequency of 1 MHz.

To further characterize the performance, power spectral density plots (PSD) of the ionic current were created for the nanopores fabricated in each device. Figure 7a presents the PSD curve of each device, and these curves clearly depict the reduced noise spectra of the polyimide device and the bead-polyimide device across the entire frequency regime. The device capacitance arising from the surface area exposed to the electrolyte solution governs the typical ionic current noise at high frequency^38^. The noise arising from the device capacitance was calculated to be 1000 pF for the bare device and 50 pF for the polyimide-coated device and bead-polyimide-coated device. The PSD spectrum of the polyimide-coated device remained nearly unchanged following coating of the bead layer. The typical current noise was highlighted by the baseline current trace of each device during the application of no voltage as illustrated in Fig. 7b. The RMS noise at 1 MHz was 580 pA_rms_ for the polyimide-coated device and 620 pA_rms_ for the bead polyimide-coated device, which represent 2.5-fold reductions (1500 pA_rms_ for the bare device). These results are consistent with previous findings that the bead layer does not affect the I-V characteristics or noise performance^33^.

## Conclusion

In summary, we have demonstrated that a single nanopore can be fabricated by the MPVI process even after coating a bead layer on the membrane. The control of the DNA translocation speed by the nanobead structure was maintained following MPVI nanopore creation. The duration of ssDNA translocation across the nanopore was decelerated by up to 10–20 μs/base, thus improving the temporal resolution of single-molecule detection. Simultaneously, the noise arising from the device capacitance was reduced by the polyimide layer. Our approach is compatible with solid-state platforms because the bead layer, polyimide layer and MPVI process did not affect performance in terms of noise, I-V characteristics, single nanopore creation or deceleration of DNA translocation. We believe that our platform will be a key component in DNA sequencing based on solid-state nanopores.

## Methods

### The bead coating and nanopore fabrication device

Substrates with 10-nm-thick Si_3_N_4_ membranes and small square areas of 500 × 500 nm^2^ were prepared (see Supplementary Figure S4) as previously reported^35^. The motion of the coated beads was confined to this square, which stabilized the bead structures. The bead coating was applied to the substrate by dip coating as previously reported^33^. Before the dip coating, the substrates were cleaned and hydrophilized on each side with argon/oxygen plasma (PA-1, SAMCO, Kyoto, Japan) at 10 W, a flow rate of 20 sccm, and a pressure of 20 Pa for 45 s. The substrates were dipped into an aqueous silica-bead solution (50-nm amine-functionalized silica beads, Micromod, Rostock, Germany) and removed at a speed of approximately 1 mm/s. After removal from the solution, the substrates were dried in an oven (DO-300PA, AS ONE, Osaka, Japan) at 120 °C for 10 minutes. The insulating polymer, i.e., polyimide, was coated on the substrate after bead coating at a thickness of 5–10 μm by spreading out a drop of polyimide under a stereoscopic microscope. The coated polyimide was cured in a thermostat chamber for 30 min at 110 °C and 30 min at 300 °C. Prior to beginning the MPVI procedure, the device was cleaned and re-hydrophilized on each side with argon/oxygen plasma at a flow rate of 20 sccm, a pressure of 20 Pa, and a power of 10 W for 45 s. The bead coating condition was confirmed by SEM (S-5200, Hitachi High-Technologies, Ibaraki, Japan) at 5 kV after the MPVI procedure and ssDNA translocation experiments.

### Nanopore fabrication by the MPVI process

MPVI was performed as follows. Two chambers (*cis* and *trans* chambers) were formed in a custom flow cell. We recently determined that an electric charge difference between the chambers generates defects in the membrane, particularly when the capacitance of the substrate is less than 100 pF^38^. To prevent initial defects, the charge difference was neutralized using an electric bypass channel connected to both chambers. Both chambers were filled with 1 M KCl aqueous solution (pH 7.5) buffered with 10 mM Tris and 1 mM EDTA. Two Ag/AgCl electrodes (*cis* and *trans* electrodes) were immersed in the solutions and connected to a pulse-voltage generator (41501B SMU and pulse generator expander, Agilent Technologies, Santa Clara, CA, USA) and an ammeter (4156B Precision semiconductor analyser, Agilent Technologies, Santa Clara, CA, USA. The MPVI procedure was controlled by a program written using Excel Visual Basic for Applications (VBA). The applied voltage and pulse duration were optimized to fabricate a nanopore with the desired diameter. The diameter of the MPVI-fabricated nanopore was calculated using equation (1).


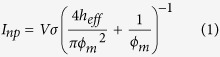


Here, *I*_np_ is the ionic current through the nanopore, *V* is the applied voltage across the membrane, *σ* is the conductivity of the solution (the measured value was approximately 10.8 S/m), *ϕ*_m_ is the diameter of the nanopore, and *h*_eff_ is the effective thickness of the membrane. We previously reported that the *h*_eff_ of a 10-nm-thick Si_3_N_4_ membrane is 3.7 nm^33^. Accordingly, we estimated the diameter of the nanopore from the relationship between *I*_np_ and *ϕ*_m_ at V = 0.1 V.

### ssDNA blockade current measurements

The prepared substrates were assembled in the same flow cell used for the MPVI method. Two chambers (a *cis* chamber and a *trans* chamber, each with a volume of 90 μL) separated by the substrate were formed in the flow cell. Both chambers were filled with an aqueous solution consisting of 1 M KCl (pH 7.5) buffered with 10 mM Tris and 1 mM EDTA. Poly(dA_60_) and poly(dA_33_dC_33_dA_33_) purchased from Nihon Gene Research Laboratory (Miyagi, Japan) were used as model ssDNA molecules. Only the solution in the *cis* chamber contained single-stranded DNA at a concentration 50 nM. An Ag/AgCl electrode was immersed in each solution to ensure electrical contact between the chambers.

To detect the ssDNA translocation events, we conducted experiments with a low-noise voltage-clamp amplifier (VC100, Chimera Instruments, New York, NY, USA). The ionic current was measured at an applied voltage of 0.3 V. The acquired data were digitally low-pass filtered at 100 kHz. Current blockade events were identified and analysed using the open-source software package OpenNanopore^40^.

## Additional Information

**How to cite this article**: Goto, Y. *et al.* Integrated solid-state nanopore platform for nanopore fabrication via dielectric breakdown, DNA-speed deceleration and noise reduction. *Sci. Rep.* **6**, 31324; doi: 10.1038/srep31324 (2016).

## Supplementary Material

Supplementary Information
