# Supplementary material for: Integrated solid-state nanopore platform for nanopore fabrication via dielectric breakdown, DNA-speed deceleration and noise reduction
Source: Sci Rep. 2016 Aug 8;6:31324. doi: 10.1038/srep31324 (PMC4976334; doi:10.1038/srep31324)
Supplement: Supplementary Information [file srep31324-s1.pdf]

Supplementary Information

Integrated solid-state nanopore platform for nanopore fabrication via dielectric breakdown, DNA-speed deceleration and noise reduction

Yusuke Goto*, Itaru Yanagi, Kazuma Matsui, Takahide Yokoi and Ken-ichi Takeda

Center for Technology Innovation - Healthcare, Research & Development Group,

Hitachi Ltd., 1-280 Higashi-Koigakubo, Kokubunji, Tokyo 185-8601, Japan

The supplementary information includes:

SI-1. Multiple nanopore fabrication under unoptimized MPVI process

SI-2. TEM image of a substrate coated with nanobeads after MPVI process

SI-3. Substrate with a membrane designed for nanopore fabrication

SI-4. Nanopore variability created by MPVI process

SI-5. Dwell time of ssDNA translocation for uncoated substrates

SI-6. Dependency of dwell time on the diameters of nanopores with beads

SI-1. Multiple nanopore fabrication under unoptimized MPVI process

We conducted the nanopore fabrication experiment under an unoptimized MPVI process (shown in Figure S1). During this MPVI process, the pulse voltage injection intentionally continued to be applied to the SiN membrane after the sudden increase of the ionic current. Figure S2 represents typical top-view TEM images of the entire area of 10-nm-thickness SiN membrane after the process. The TEM images confirm at a glance that three nanopores with diameters of 3 – 5 nm were simultaneously fabricated in the SiN membrane. The result clearly indicated that unoptimized breakdown process induced creation of multiple nanopores. Note that this phenomenon might be specific to the breakdown process when using MPVI.

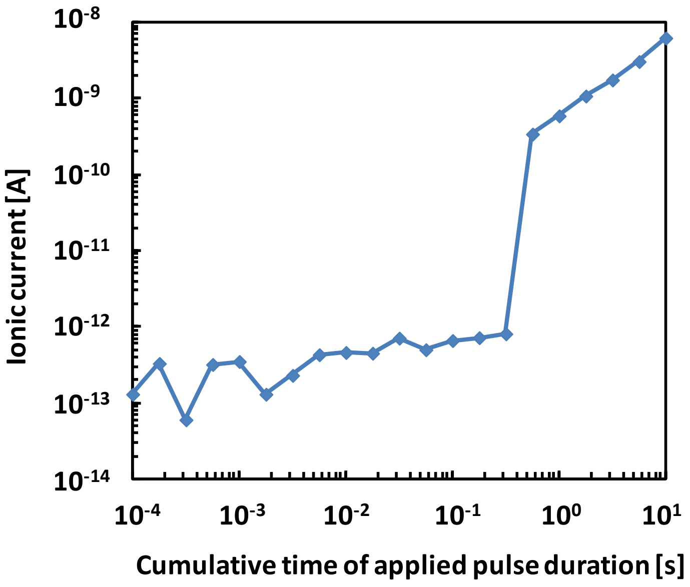

Figure S1. Dependence of ionic current at 0.1 V on the cumulative time of applied-pulse durations during unoptimized MPVI process. The lot of SiN membrane used in this experiment has the breakdown voltage of 6 V (The different lot used in the main text has the breakdown voltage of 9 V.). During the process, the same 6 V voltage continued to be applied to the SiN membrane until the ionic current reached at 6 nA.

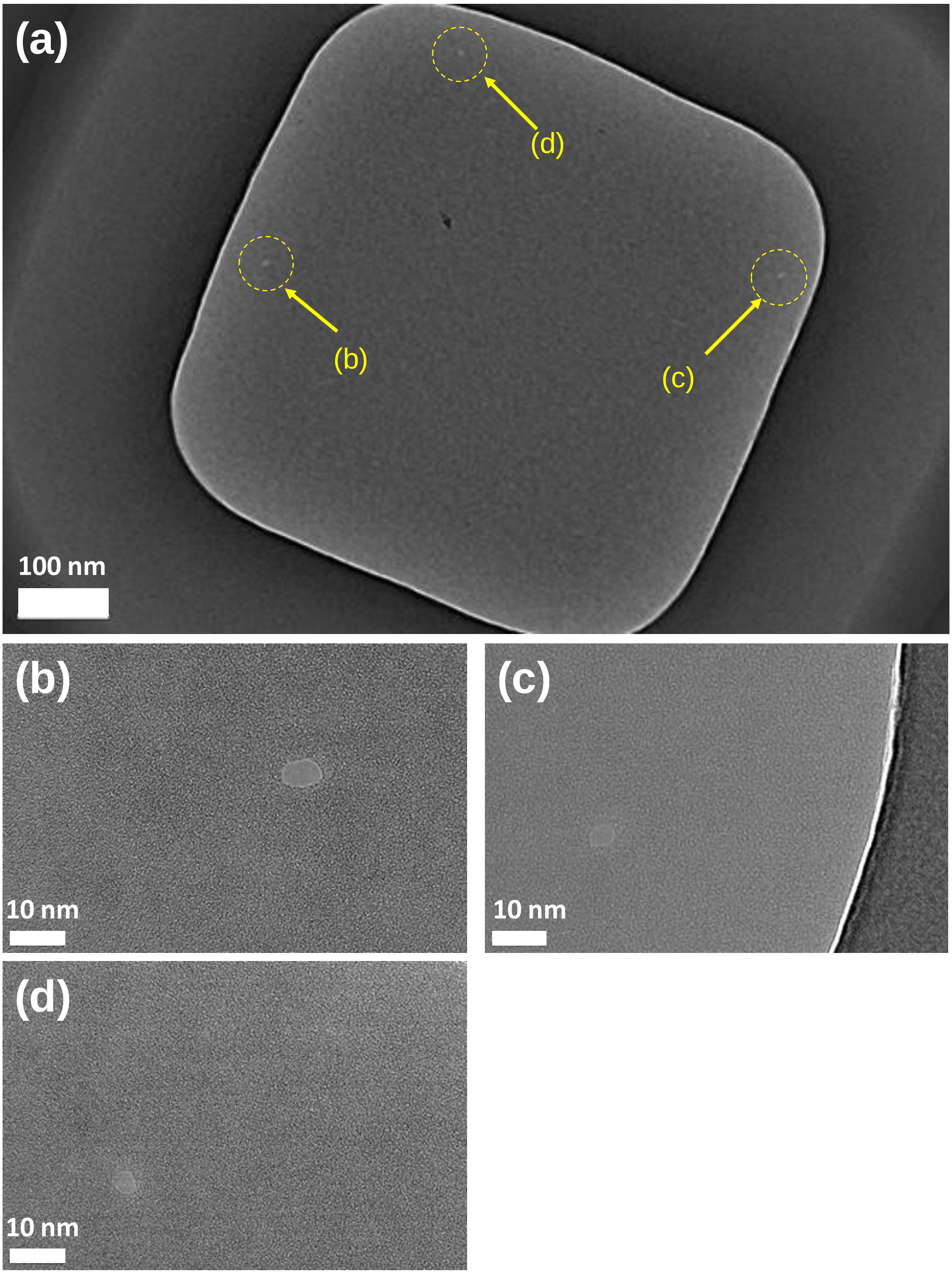

Figure S2. (a) Top-view TEM image of the entire area of 10-nm-thickness SiN membrane after unoptimized MPVI process. (b)-(d) Magnified views of nanopores shown in (a). TEM images confirm that nanopores with diameters of 3 – 8 nm were fabricated.

SI-2. TEM image of a substrate coated with nanobeads after MPVI process

A membrane of a substrate was observed using TEM (JEM-2100F, JEOL, Ltd., Tokyo, Japan) at an accelerating voltage of 200 kV. Before the observation, the device was washed with pure water to remove any residual salts from the solution. Figure S3 shows a typical TEM image of the substrate coated with nanobeads after nanopore fabrication via MPVI process. To clearly observe the membrane by TEM, the number of beads on the membrane was reduced. The observation revealed that a nanopore cannot be confirmed using TEM because the beads prevented an electronic beam.

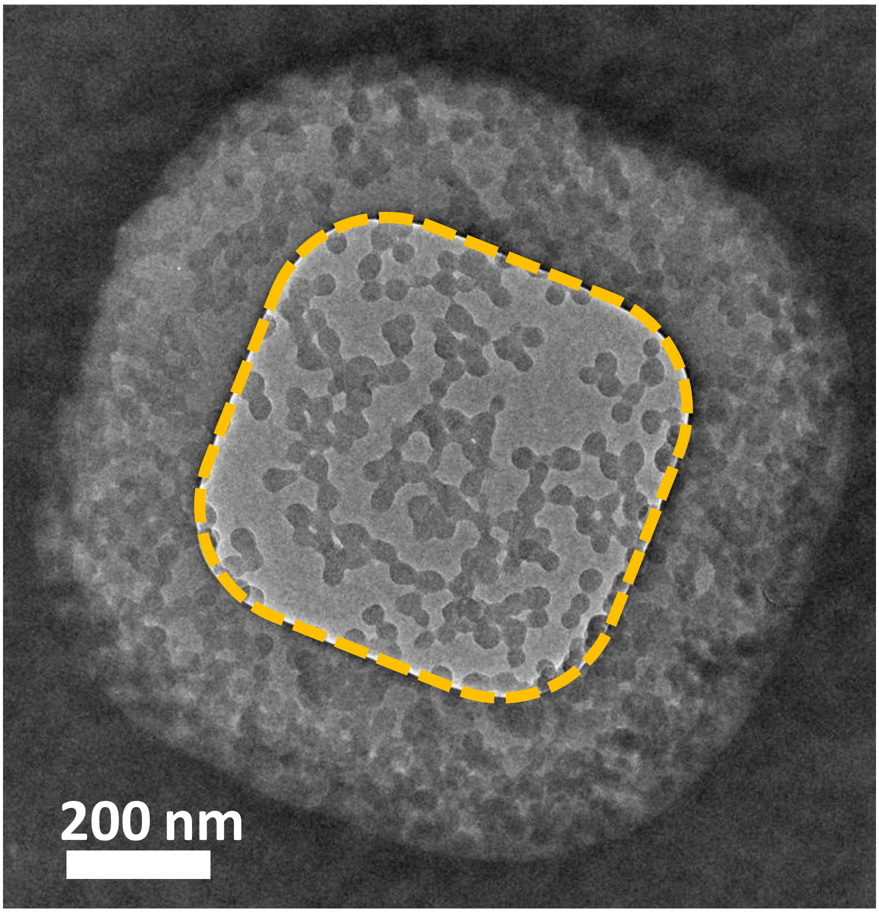

Figure S3. Top-view TEM image of the substrate coated with nanobeads after MPVI process. The square area is surrounded by the dashed line (orange).

SI-3. Substrate with a membrane designed for nanopore fabrication

Figure S4 shows schematic image of a substrate with a membrane for nanopore fabrication, as previously reported [1]. Bead layer and polyimide layer are coated on the substrate as described in the main text.

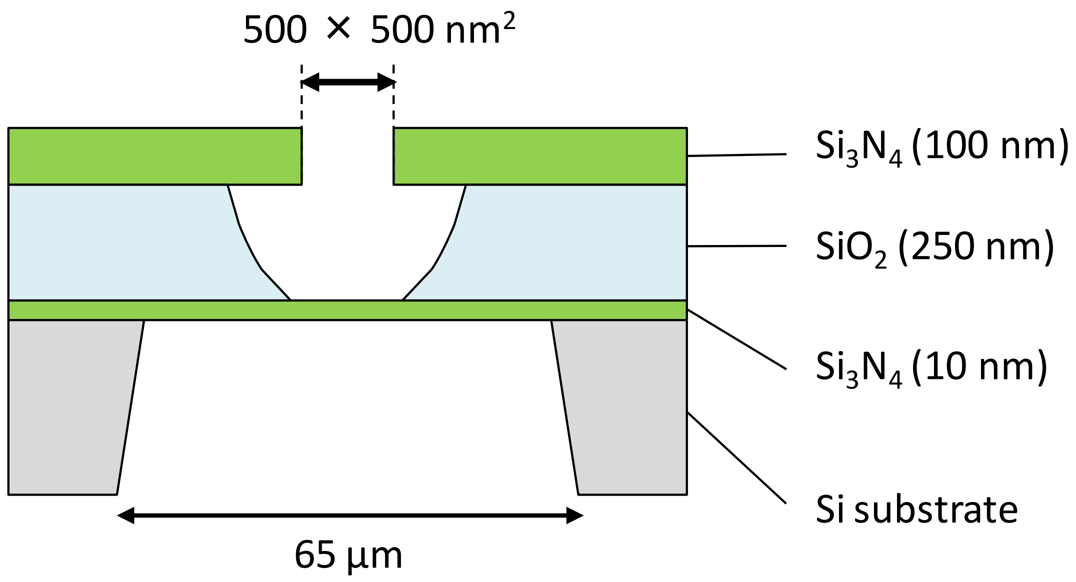

Figure S4. Substrate with a membrane designed for nanopore fabrication. A 100-nm-thick Si_3N_4 layer, a 250-nm-thick SiO_2 layer, and a 10-nm Si_3N_4 layer were deposited on Si substrates. A square hole of 500 × 500 nm^2 was fabricated by dry etching, and the SiO_2 layer was partially eliminated by HF etching.

SI-4. Nanopore variability fabricated by MPVI process

Table S1 represents typical nanopore variability data (N = 15) fabricated by MPVI process after optimization of parameters. The target current value is preset as 340 pA at 0.1 V, corresponding to 1.4-nm-diameter nanopore. MPVI process can fabricated the nanopores with diameter of 1.6 ± 0.15 nm, whose variation was quite small. This statistical data shows that MPVI can fabricate precisely and reproducibly a single nanopore.

Table S1. Nanopore variability data (N=15) fabricated by MPVI process. All nanopores were fabricated using the substrate possessing the 10-nm-thickness SiN membrane with beads and polyimide layer. I_{np} is the ionic current at 0.1 V and ϕ_m is the predicted diameter of the nanopore.

No	I_{np} (pA @ 0.1 V)	ϕ_m [nm]
1	393	1.53
2	342	1.41
3	556	1.87
4	352	1.44
5	402	1.55
6	459	1.67
7	347	1.43
8	452	1.66
9	506	1.77
10	431	1.61
11	557	1.87
12	345	1.42
13	483	1.72
14	464	1.68
15	401	1.55

SI-5. Dwell time of ssDNA translocation for uncoated substrates

We investigated the dwell time of short ssDNA translocation for uncoated substrates. Figure S5 depicts typical log-scaled histograms of the dwell time for 60-mer poly(dA) passing through the 1.7-nm-diameter and 2.2-nm-diameter nanopores. The histograms were well-fitted to a single log-normal distribution and the characteristic dwell times (the peak position of the fitted curve) were 40 μs (1.7 nm) and 20 μs (2.2 nm), respectively. The obtained dwell time is sufficient longer than twice the filter rise time (2*Tr = 2*0.332/ f_c = 6.6 μs at f_c = 100 kHz) [2]. This result indicated that the measured events were not attenuated by the filter. Therefore, the obtained ssDNA translocation speeds were calculated as 0.6 μs/base (1.7 nm) and 0.3 μs/base (2.2 nm), which were consistent with the previsous reported values [3][4].

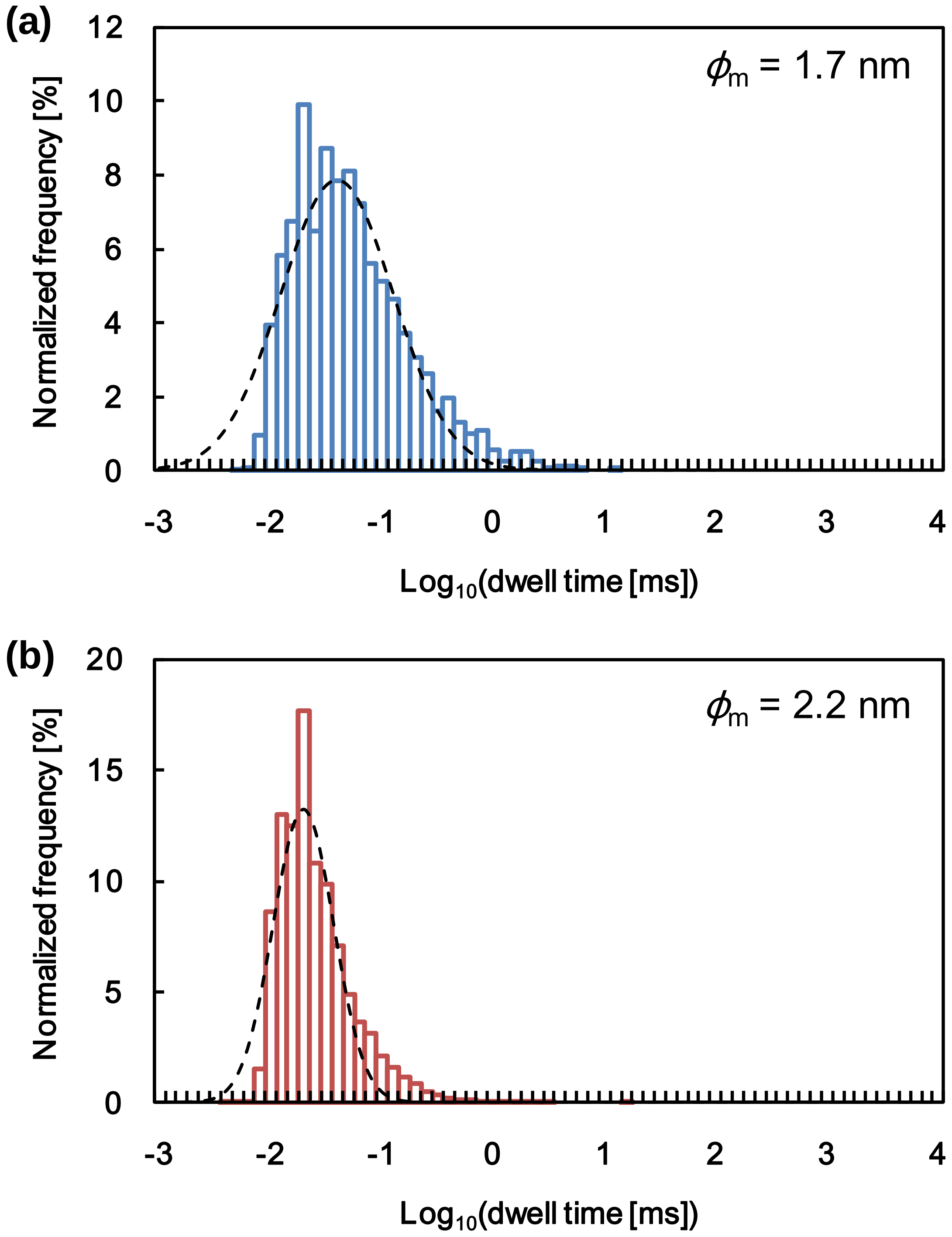

Figure S5. Log-scaled histograms of dwell times for 60-mer poly(dA) passing through nanopores using uncoated substrates. Nanopore diameters were (a) 1.7 nm (N = 3547) and (b) 2.2 nm (N = 3643). The data was measured at 0.3 V and digitally filtered at 100 kHz filter. Broken lines were fitted curves using a log-normal distribution.

SI-6. Dependency of dwell time on the diameter of the nanopore with beads

To investigate the dependence of the dwell time on the diameter of the nanopore with nanobeads, we conducted the 60-mer ssDNA translocation experiments using nanopores with other diameters of 1.6 nm and 2.3 nm. Figure S6 represents the log-scaled histograms of the dwell time for 60-mer poly(dA) passing through nanopores. Similarly to Figure S5, both histograms were well-fitted by a single log-normal distribution and the characteristic dwell times were 620 μs (1.6 nm) and 530 μs (2.3 nm), respectively. Accordingly, the translocation speed of ssDNA was calculated to be 10.2 μs/base (1.6 nm, 60-mer ssDNA), 8.9 μs/base (2.3 nm, 60-mer ssDNA), 16 μs/base (3.3 nm and 3.7 nm, 60-mer ssDNA, the same data shown in Figure 6a) and 22 μs/base (2.2 nm and 3.4 nm, 99-mer ssDNA, the same data shown in Figure 6b), indicating that the translocation speed is almost independent on the nanopore's diameter when using bead-coated substrate (Figure S7). Compared with nanopores for the uncoated substrate as shown in Figure S5, ssDNA translocated more slowly through nanopores for bead-coated substrate at the average speed of 16 μs/base. These results supports that the bead layer can decelerate ssDNA translocation speed.

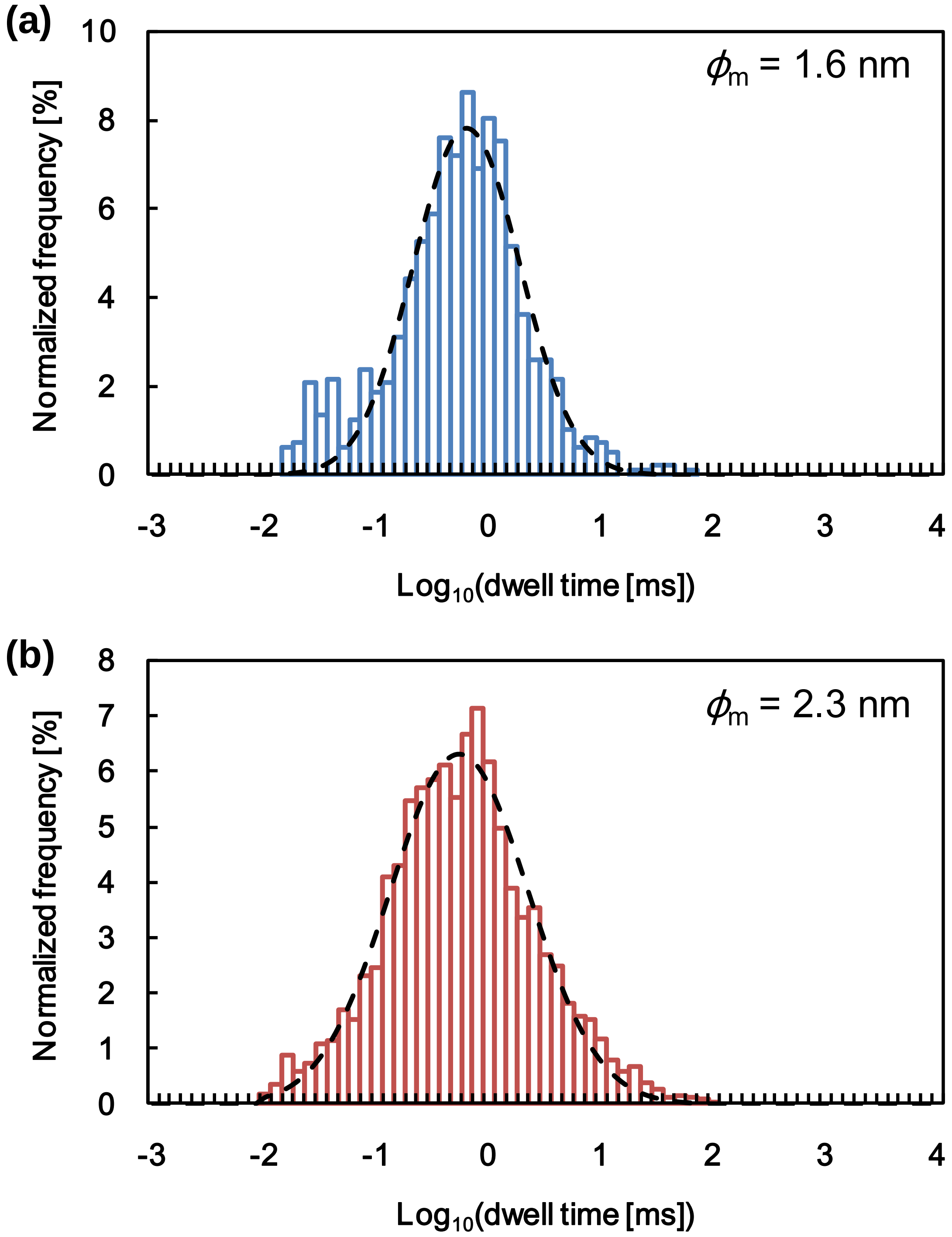

Figure S6. Log-scaled histogram of dwell time for 60-mer poly(dA) passing through nanopores using bead and polyimide layer-coated substrates. The data was measured using the nanopores with the diameter of (a) 1.6 nm (blue, N = 2043) and (b) 2.3 nm (red, N = 7281). The data was measured at 0.3 V and digitally filtered at 100 kHz filter. Broken lines were fitted curves using a log-normal distribution.

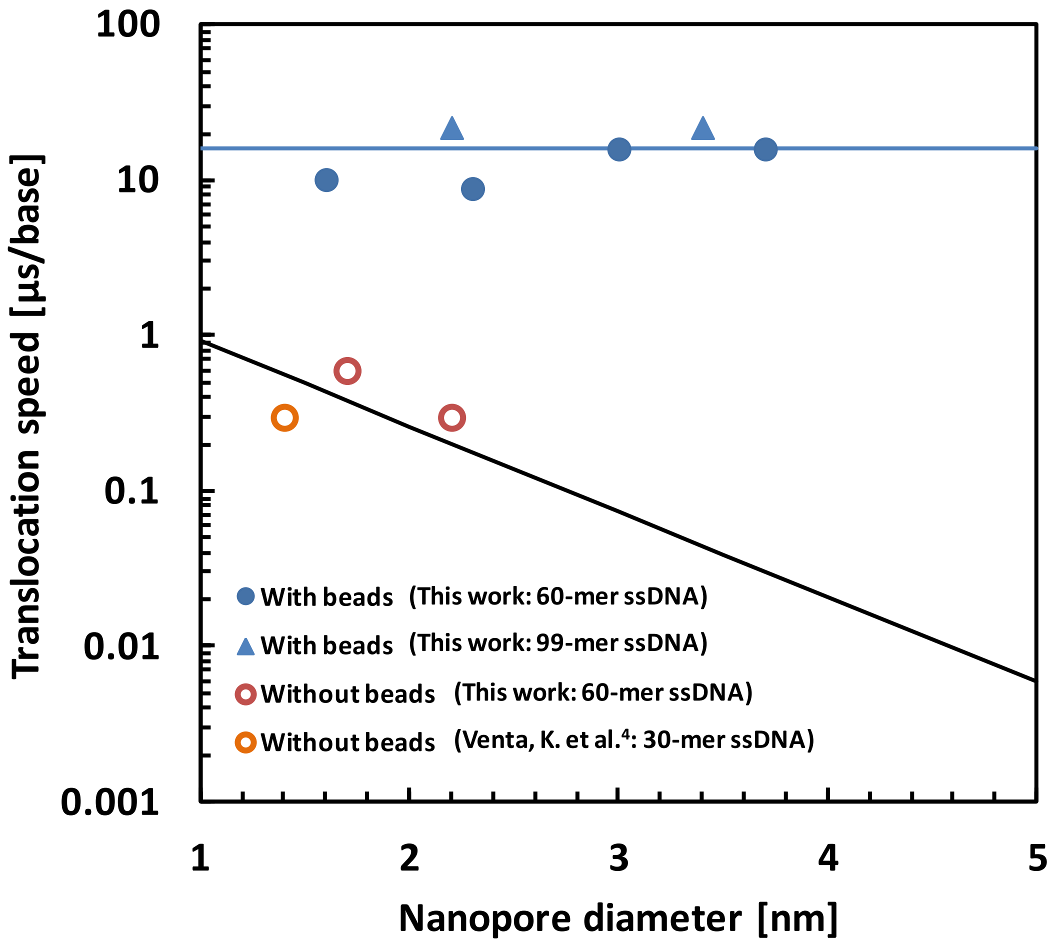

Figure S7. Short single-stranded DNA translocation results as a function of SiN nanopore diameter with or without nanobeads. Translocation speed of ssDNA results from our own experiments plotted along with the literature data. The data were extracted directly from the literature. All measurements were performed in 1M KCl electrolyte. Blue line is the average speed of the results with beads. Black line is a power approximation to the results without beads predicted from the literature data by Akahori, et al.[3] using long 5300-mer ssDNA.

References:

[1] Yanagi, I., Akahori, R., Hatano, T. & Takeda, K. Fabricating nanopores with diameters of sub-1 nm to 3 nm using multilevel pulse-voltage injection. *Sci. Rep.* **4**, 5000 (2014).

[2] Plesa, C. *et al.* Fast translocation of proteins through solid state nanopores. *Nano Lett.* **13**, 658-663. (2013).

[3] Akahori, R. *et al.* Slowing single-stranded DNA translocation through a solid-state nanopore by decreasing the nanopore diameter. *Nanotechnology* **25**, 275501 (2014).

[4] Venta K., *et al.* Differentiation of Short, Single-Stranded DNA Homopolymers in Solid-State Nanopores. *ACS Nano* **7**, 4629-4636 (2013).
